# Supplementary material for: Millennial scale persistence of organic carbon bound to iron in Arctic marine sediments
Source: Nat Commun. 2021 Jan 12;12:275. doi: 10.1038/s41467-020-20550-0 (PMC7804933; doi:10.1038/s41467-020-20550-0)
Supplement: Supplementary file 1 — Supplementary Information [file 41467_2020_20550_MOESM1_ESM.pdf]

Supplementary Information

Millennial scale persistence of organic carbon bound to iron in Arctic marine sediments

Faust et al.

Supplementary Figures S1-S7

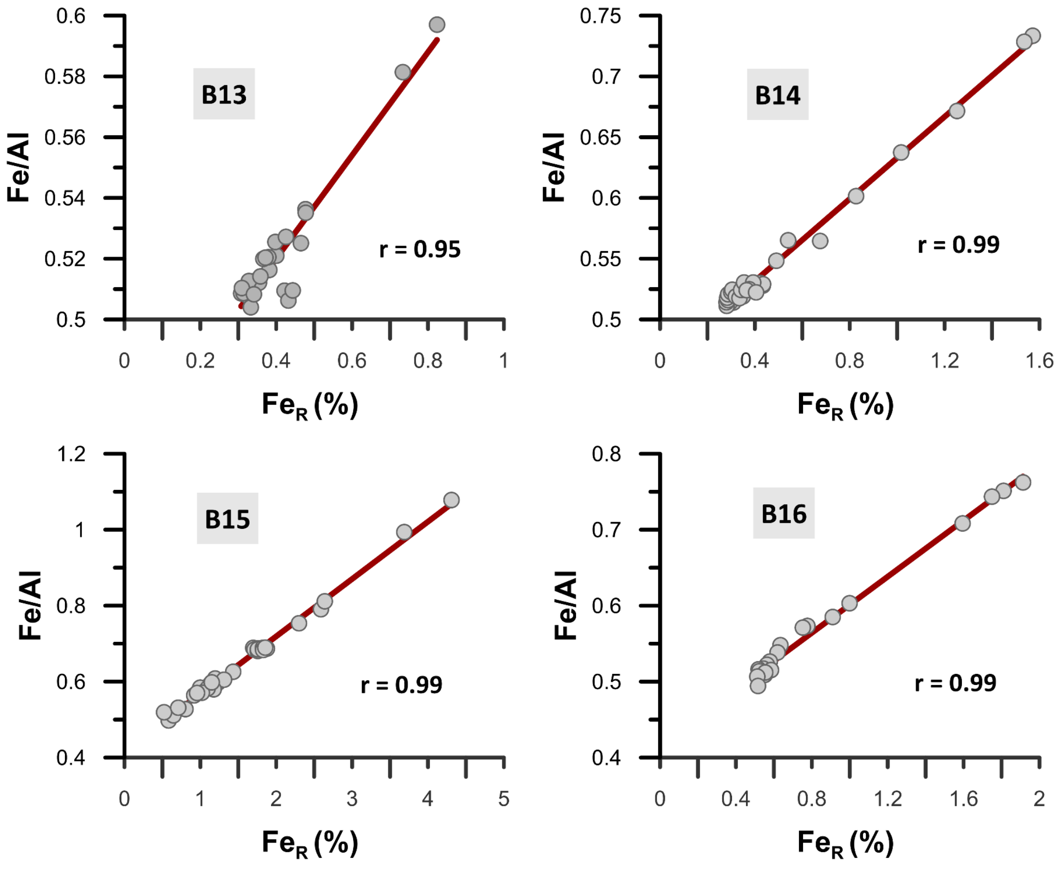

Supplementary Fig. S1: Correlation between Fe/Al and reactive iron (Fe_R) in all four sediment cores investigated in this study.

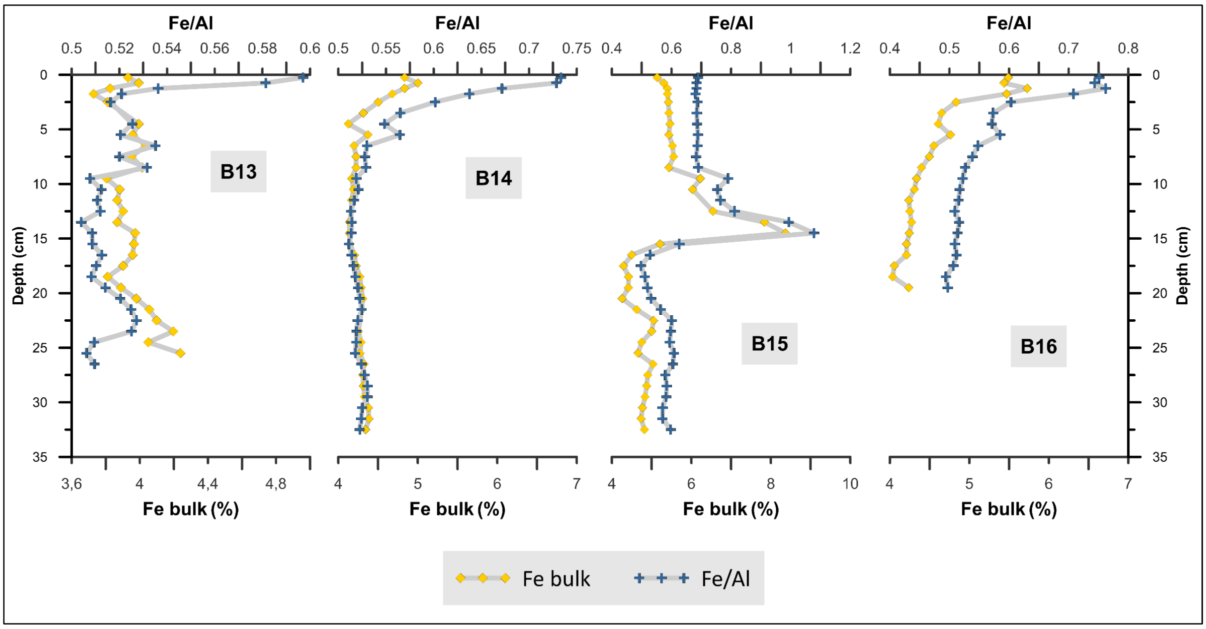

Supplementary Fig. S2: Downcore profiles of bulk iron content (Fe) and Fe/Al in all four sediment cores investigated in this study.

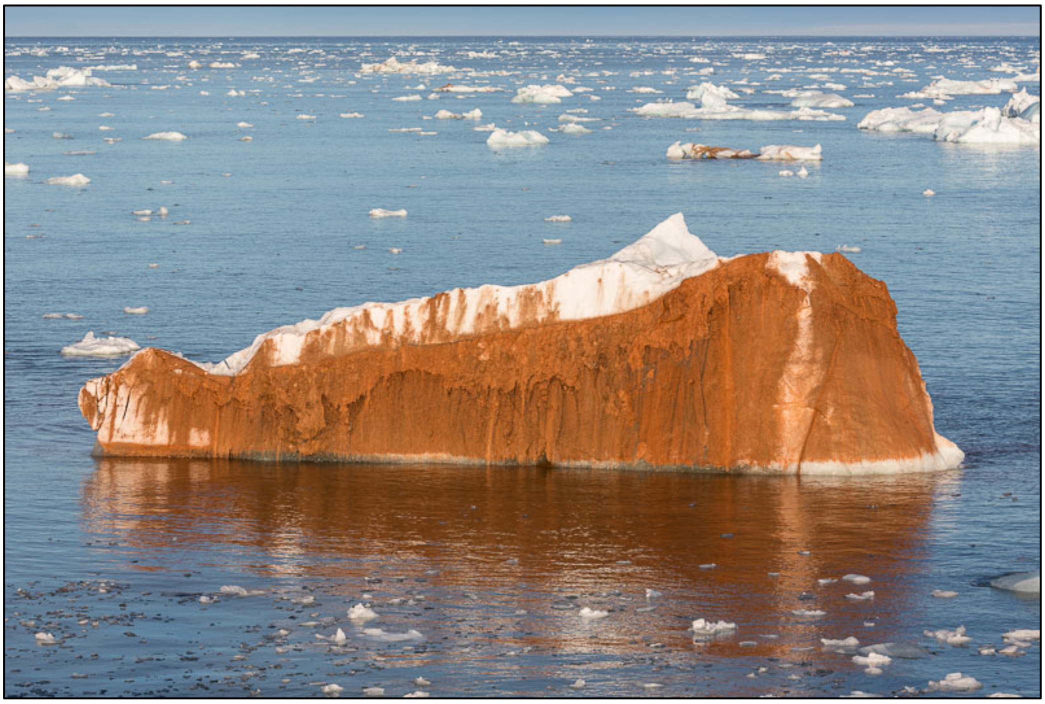

Supplementary Fig. S3: Iceberg covered in reddish sediment. Picture was taken on the 21st of July 2016 near the front of the ice stream draining from Austfonna on Nordaustlandet, which is the largest icecap in Svalbard. Copyright: J. Alean (https://www.swisseduc.ch).

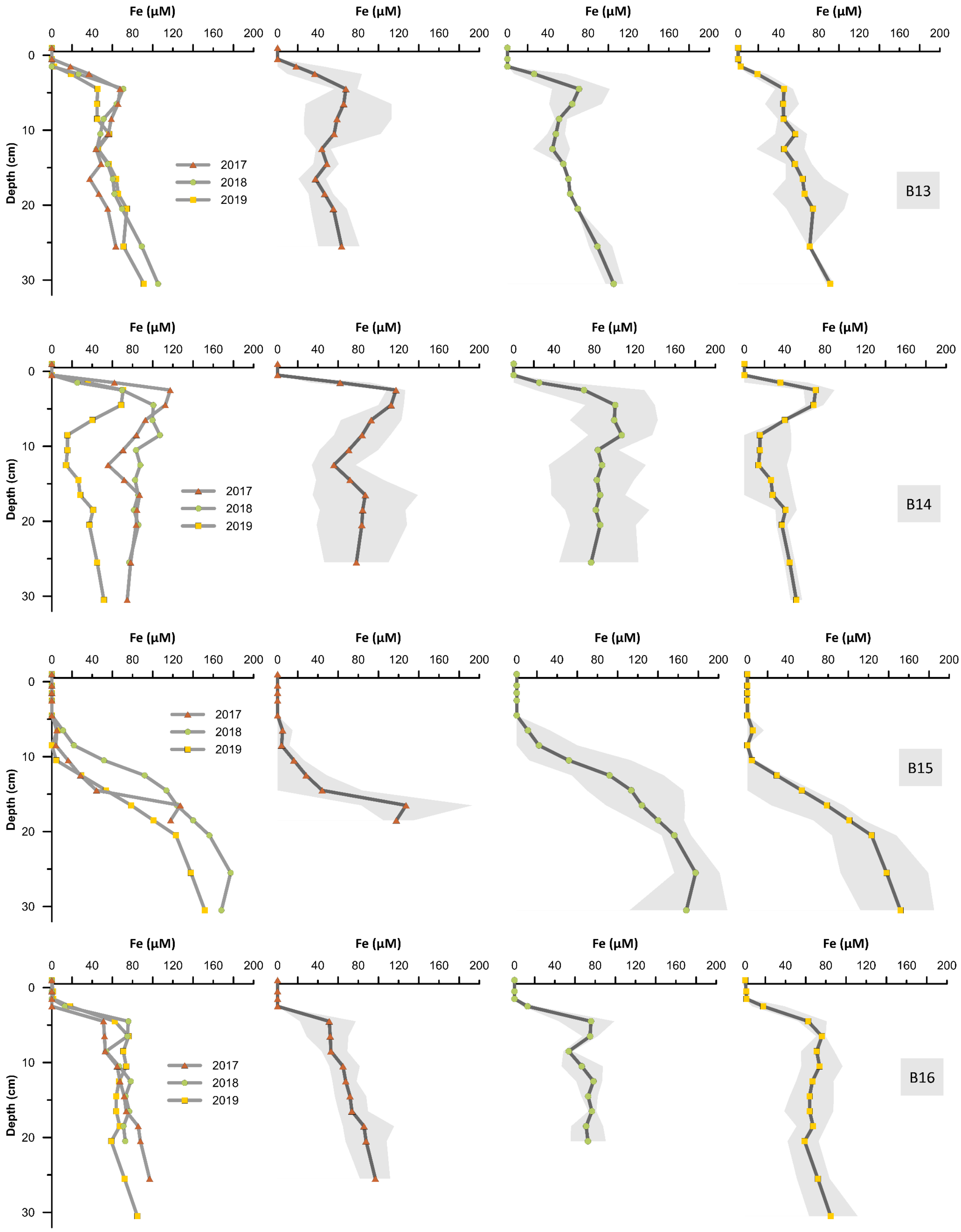

Supplementary Fig. S4: Comparison of average downcore iron porewater profiles from our sampling campaigns in summer 2017 (red triangle), 2018 (green circles) and 2018 (yellow squares). The solid grey area indicates maximum and minimum pore water iron concentration from three different cores at the same coring location collected each year.

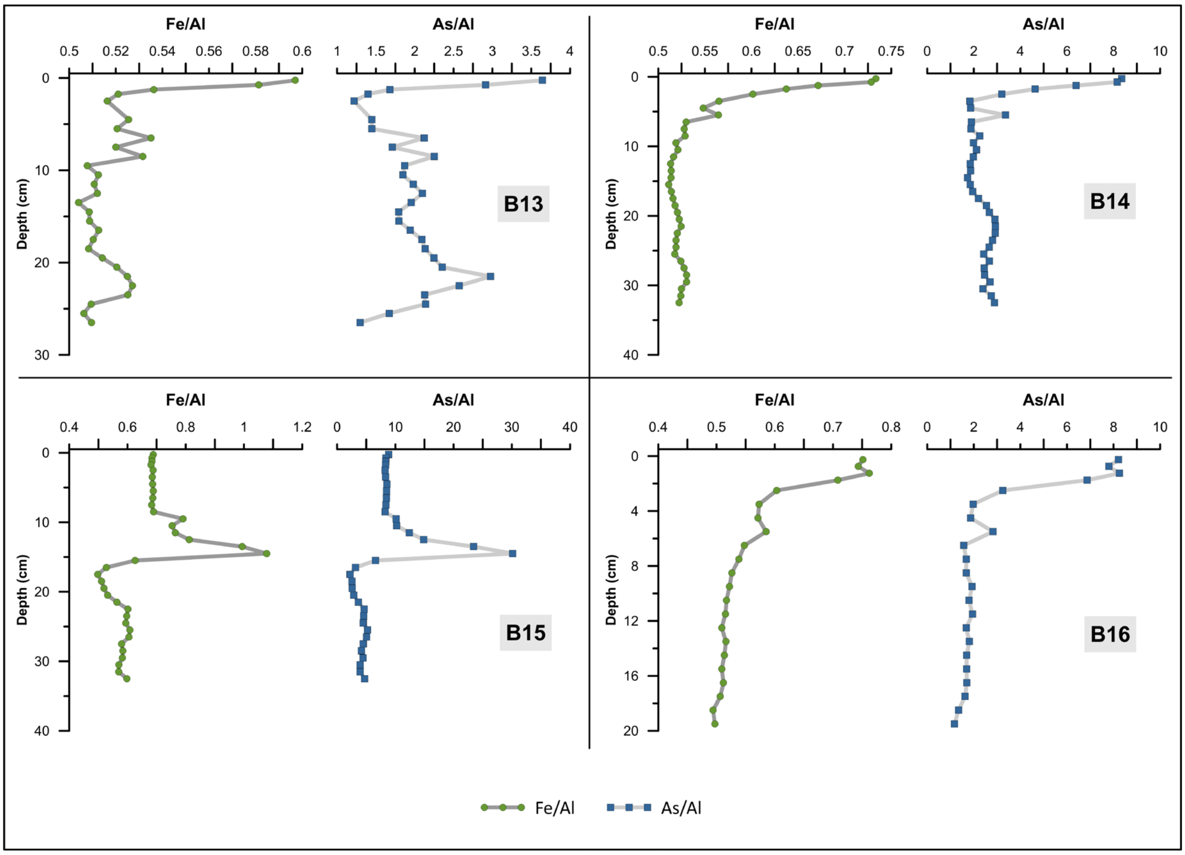

Supplementary Fig. S5: Downcore profiles of Fe/Al (green circles) and As/Al (blue squares).

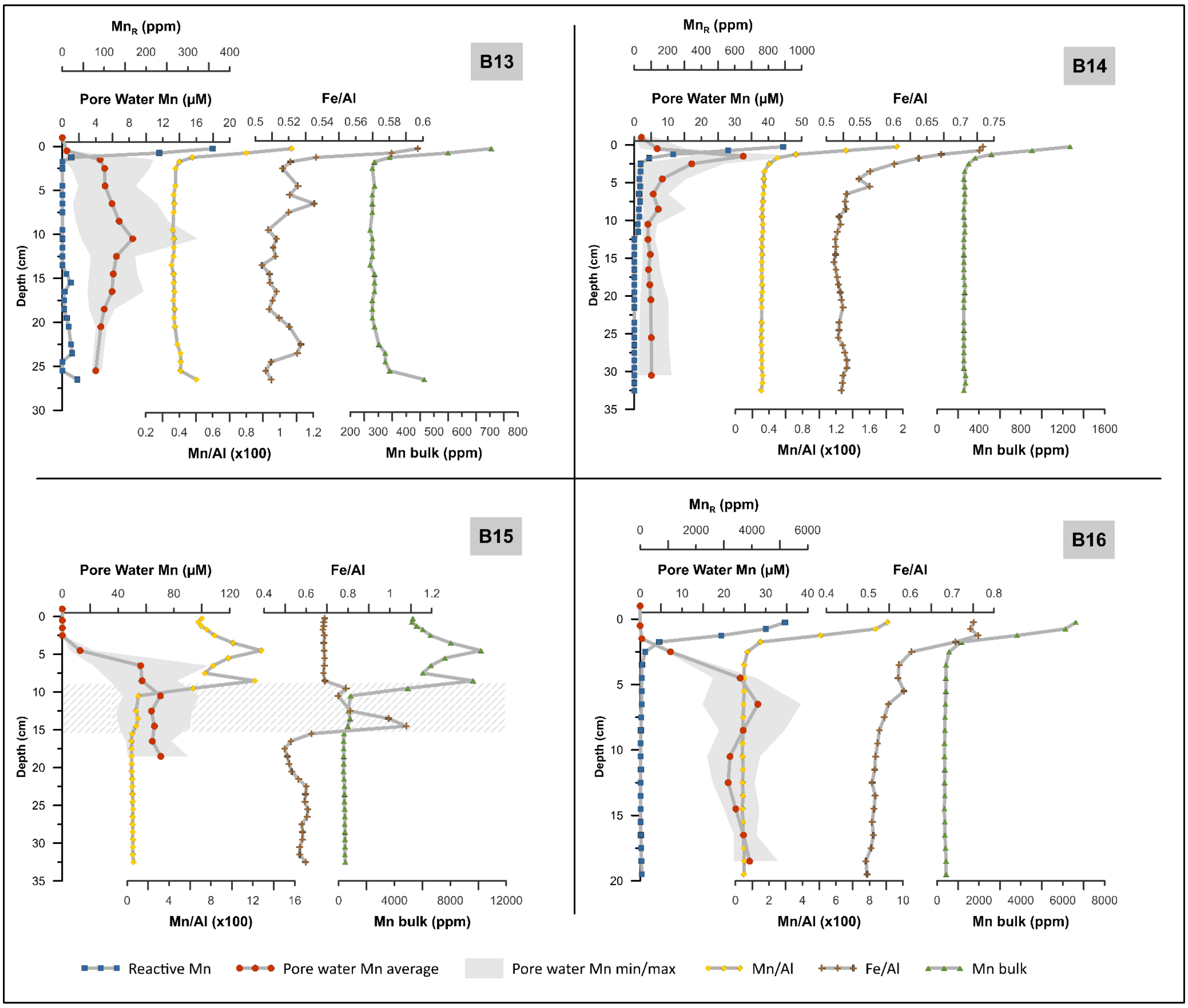

Supplementary Fig. S6: Downcore measurements of pore water manganese content and sedimentary Mn/Al, reactive manganese (Mn_R), Fe/Al and bulk manganese contents. The solid grey area indicates maximum and minimum pore water manganese concentration measured on three different cores at the same coring location. The shaded grey area at station B15 marks the location of a reddish/pink sediment layer. Note the different scale of the x-axis for each core and Mn_R is not available due to an instrumental failure at station B15.

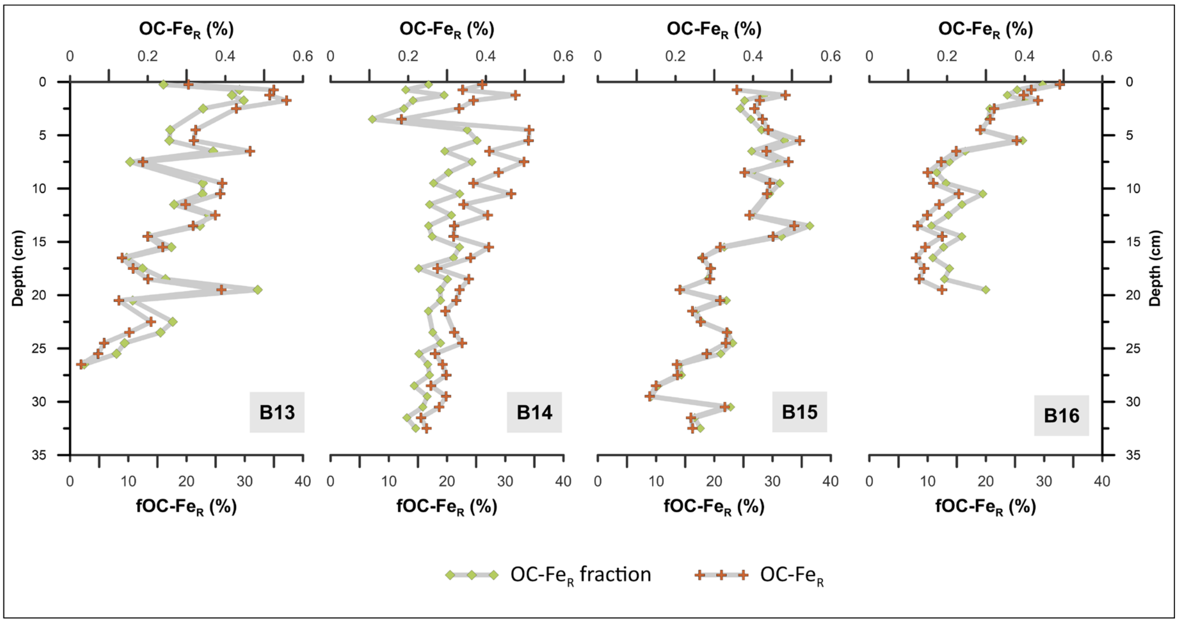

Supplementary Fig. S7: Downcore profiles OC-Fe_R versus fOC-Fe_R.

Supplementary Notes

Further details of the organic carbon (OC) and reactive iron analysis (Fe_R).

To quantify the amount of OC bound to Fe_R and Mn_R, we applied the extraction method described in detail by Lalonde et al. [1] and ‚Salvadó et al. [2]. Consequently, we used the control extractions to quantify the extracted OC which is not associated with Fe_R or Mn_R. The results of the individual control experiments were subtracted from the amount of OC released from the dithionite extraction. Thus, all data were control-corrected in order to obtain reliable OC-Fe values. Additionally, to account for the mass loss during the extraction experiment we applied the mass balance calculation of Salvado et al. [2]. Equation 1 was used to determine %OC-Fe_R:

$$\text{OC-Fe}_R\ (\%) = 100\cdot(OC_{control} - OC_{after})\cdot(M_{control}/M_{sample})/(OC_{initial}) \quad (1)$$

OC-Fe_R (%) = Percentage of OC attached to reactive iron.
$TOC_{control}$ = Total organic carbon content after the control extraction.
TOC_{after} = Total organic carbon content after the reduction extraction.
$TOC_{initial}$ = Total organic carbon content of the bulk sediment.
$M_{control}$ = Mass before the control extraction.
M_{sample} = Mass before the reduction extraction.

Note that liquid-HCl decarbonation of the bulk sediment samples may also dissolve reactive iron phases in addition to carbonates, which potentially could liberate some iron-bound OC, which would bias our bulk organic carbon results to lower values. Our reported OC-Fe_R values might therefore be biased towards slightly higher values due to a downward bias in the denominator of Eq. 1.

The OC fraction of the total OC bound to Fe_R (fOC-Fe_R) was calculated from the TOC_{inital} and OC-Fe_R content for each individual sample.

Dissolved iron and manganese in the supernatant of the reduction experiments are assumed to represent Fe_R and Mn_R. Iron and manganese liberated during the control experiment were subtracted from the dithionite extractable iron and manganese contents, however, iron values were negligibly small (<0.025%) and all manganese measurements were below the detection limit.

The fraction of Fe_R of total iron (fFe_R) was calculated from the bulk Fe content and the Fe_R content for each individual sample.

References

1 Lalonde, K., Mucci, A., Ouellet, A. & Gelinas, Y. Preservation of organic matter in sediments promoted by iron. *Nature* **483**, 198-200 (2012).

2 Salvadó, J. A. *et al.* Organic carbon remobilized from thawing permafrost is resequestered by reactive iron on the Eurasian Arctic Shelf. *Geophysical Research Letters* **42**, 8122-8130 (2015).
