# Supplementary material for: Millennial scale persistence of organic carbon bound to iron in Arctic marine sediments
Source: Nat Commun. 2021 Jan 12;12:275. doi: 10.1038/s41467-020-20550-0 (PMC7804933; doi:10.1038/s41467-020-20550-0)
Supplement: Supplementary file 3 — Description of Additional Supplementary Files [file 41467_2020_20550_MOESM3_ESM.pdf]

Description of Additional Supplementary Files

File: Supplementary Data 1

Description: Sampling location, pore water data as well as OC-Fe results and the elemental composition of the sediment samples shown and discussed in our manuscript.
